# A new specimen of *Solnhofia parsonsi* from the Upper Jurassic (Kimmeridgian) Plattenkalk deposits of Painten (Bavaria, Germany) and comments on the relationship between limb taphonomy and habitat ecology in fossil turtles

**DOI:** 10.1371/journal.pone.0287936

**Published:** 2023-07-26

**Authors:** Felix J. Augustin, Márton Rabi, Frederik Spindler, Panagiotis Kampouridis, Josephina Hartung, Raimund Albersdörfer, Andreas T. Matzke

**Affiliations:** 1 Department of Geosciences, University of Tübingen, Tübingen, Germany; 2 Natural Sciences Collections, Martin Luther University Halle-Wittenberg, Halle, Germany; 3 Dinosaurier Museum Altmühltal, Denkendorf, Germany; University of Silesia, POLAND

## Abstract

The limestones of the Solnhofen area in southern Germany are one of the most important fossil Lagerstätten from the entire Mesozoic era, especially famous for the exquisitely preserved vertebrates. The turtles from the Solnhofen Limestone have been always of special interest because they include some of the best-preserved specimens from the Mesozoic. Here, we describe a new turtle specimen from the Torleite Formation (Kimmeridgian) of Painten and refer it to the thalassochelydian turtle *Solnhofia parsonsi* based on the presence of a unique combination of characters. The far majority of morphological differences from previously published specimens can be explained by ontogeny as the new specimen represents a larger, more ossified, and presumably older individual. Additionally, the specimen from Painten is the first described specimen of *S*. *parsonsi* preserving the largely complete and articulated limbs, the preservation of which indicates that the taxon did not possess stiffened paddles present in more pelagic marine turtles and is consistent with a previously inferred nearshore marine lifestyle. Contrary to previous inferences, we argue that taphonomic preservation of digits in articulated fossil turtles from laminated deposits cannot be used alone to infer marine or freshwater habitat. Finally, the new specimen from Painten is only the second, for which detailed information on its stratigraphic position and locality of origin are known.

## Introduction

The Upper Jurassic laminated limestone (German Plattenkalk) of the Franconian Alb in southern Germany represents one of the most important fossil Lagerstätten of the entire Mesozoic era. Especially the exquisitely preserved vertebrates, many with preserved soft tissues, made these deposits world famous, which are also known as the Solnhofen limestone, named after one of the most important localities on the Franconian Alb. Aside from abundant and diverse fishes–certainly the most common vertebrate group–the Plattenkalk of southern Germany yielded the remains of many different tetrapod clades, including ichthyosaurs, plesiosaurs, squamates, crocodyliforms, turtles, pterosaurs, non-avian theropod dinosaurs, and basal avialans. Among these groups, the turtles of the Solnhofen limestone and similar deposits in southwestern Germany, Switzerland and France have become the focus of detailed studies recently, concerning both their taxonomy and their palaeoecology (e.g., [1–6]). Despite this growing interest, many important turtle specimens that have been unearthed in recent years still await detailed study. This material, including some of the best-preserved turtle specimens recovered thus far, is, however, crucial for a better understanding of the diversity and ecology of the turtles from the Plattenkalk deposits of Europe.

**Fig 1:**
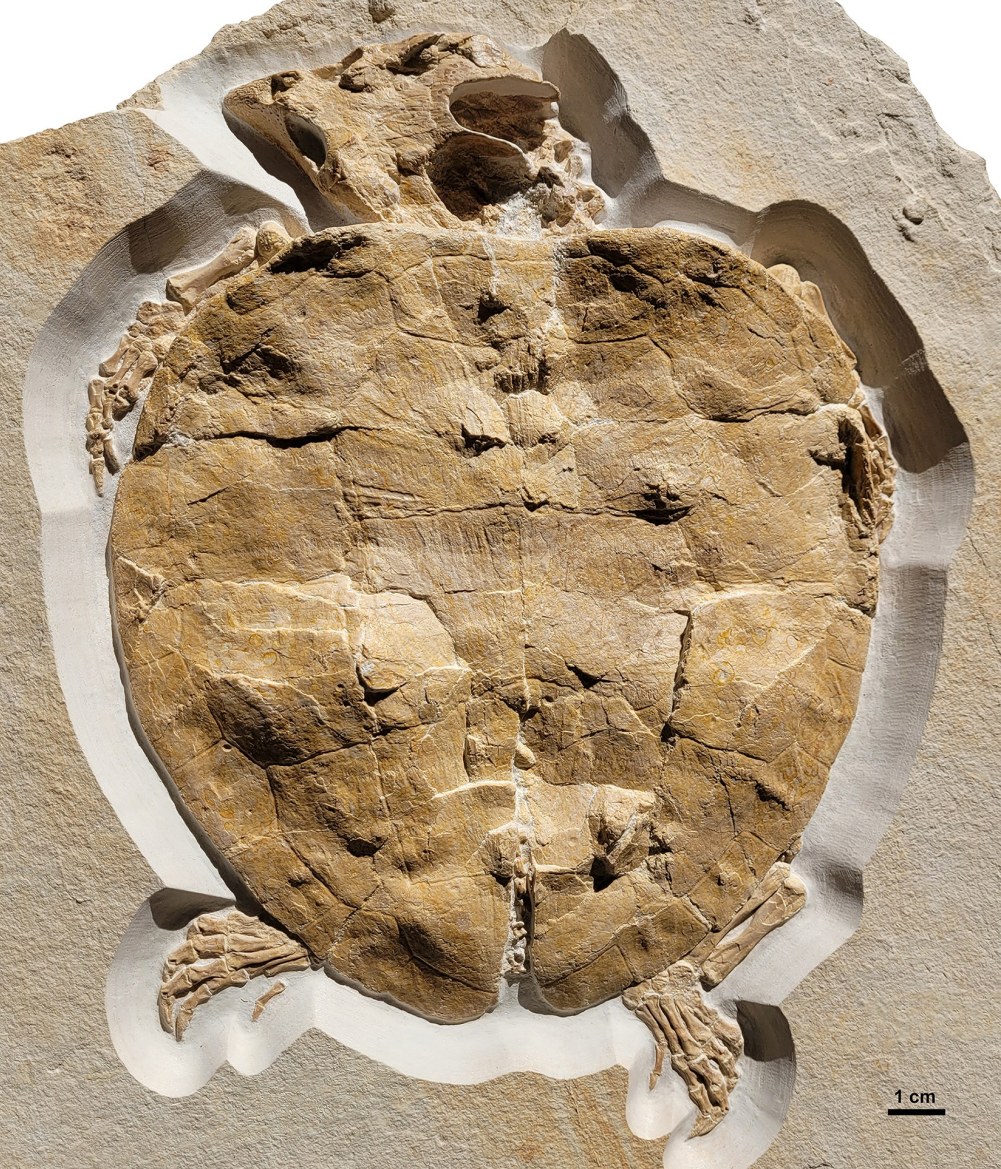
*Solnhofia parsonsi*, DMA-JP-2004/005, from the Upper Jurassic (Kimmeridgian) Torleite Formation of Painten. Overview photograph of the specimen in dorsal view.

Such important new turtle specimens, currently lacking detailed study, are known primarily from the Plattenkalk deposits of the Rygol Quarry near Painten, located on the southeastern Franconian Alb. Although this quarry has been operated since the 1950s, systematic excavations have only relatively recently been conducted, first by Wolfgang Haeckel and later by the private Albersdörfer institute [7]. These excavations have yielded a diverse vertebrate fauna [7–11], including several exquisitely preserved, and yet to be described turtle specimens [10]. A recent examination of the fossil vertebrate collection from the Plattenkalk of Painten, housed in the Dinosaurier Museum Altmühltal (DMA), also led to the examination of DMA-JP-2004/005, an exquisitely preserved turtle specimen that has provisionally been referred to as *Solnhofia* sp. (Fig 1). In this paper, a detailed description of this specimen is provided and, based on a thorough comparison with the two previously recognized species assigned to the genus *Solnhofia*, it is assigned to *Solnhofia parsonsi*.

The genus *Solnhofia*, with the type species *S*. *parsonsi*, was erected by Gaffney (1975) [12] based on two nearly complete skulls from the Upper Jurassic of Bavaria and Solothurn (Switzerland). A third specimen of *S*. *parsonsi* comprising the skull and an associated postcranium from Schamhaupten (Bavaria) has subsequently been referred to this species [13]. Additional cranial and postcranial material from the Upper Jurassic of France was later referred to *Solnhofia* sp. and *Solnhofia* cf. *parsonsi* [14–16], although the taxonomic affinities of this material are not entirely clear [2, 15]. A second species, *S*. *brachyrhyncha*, was erected recently and was based on a largely complete skull and associated postcrania from Solothurn, Switzerland [4]. Recent phylogenetic analyses have consistently placed *Solnhofia* in the *Thalassochelydia* [6, 17, 18], a group of predominantly coastal marine turtles from the Upper Jurassic and Lower Cretaceous of Europe and South America [2]. Moreover, based on morphological comparisons, *Solnhofia* has also been suggested to belong to the *Sandownidae* [19], a group that is likely part of Thalassochelydia [4, 6, 17, 19].

### Institutional abbreviations

BMM, Bürgermeister-Müller-Museum, Solnhofen, Germany; DMA, Dinosaurier Museum Altmühltal, Denkendorf, Germany; JM, Jura Museum, Eichstätt, Germany; MJSN, Jurassica Museum, Porrentruy, Switzerland; NMS, Naturmuseum Solothurn, Switzerland; TM, Teyler Museum, Haarlem, Netherlands.

## Geological setting

The specimen described herein was discovered near Painten on the southeastern part of the Franconian Alb in central Bavaria, southeastern Germany. The Franconian Alb forms, together with the Swabian Alb (its western continuation in the state of Baden-Württemberg) a low mountain range, which extends from the southwest to northeast in southern Germany and is composed of Lower to Upper Jurassic marine sedimentary rocks. Several uppermost Jurassic localities of the Franconian and Swabian Alb have yielded abundant and exceptionally well-preserved fossils of plants, invertebrates and vertebrates. These sites are generally characterized by an extremely fine-grained, laminated and planar limestone usually lacking bioturbation, which is often also called by its German name ‘Plattenkalk’ [20]. Perhaps the most significant fossil localities, especially in terms of historical importance, are located in the southern part of the Franconian Alb near Solnhofen and Eichstätt, although several other sites of this region yielded a rich fossil assemblage with a similar preservation as well, including (from southwest to northeast) Daiting, Schamhaupten, Painten, Kelheim, Jachenhausen, Zandt and Brunn. Together, the Plattenkalk deposits of the southern Franconian Alb form the ‘Solnhofen archipelago’, also referred to as the ‘Solnhofen limestone’. Two other important fossil sites preserving a similarly rich and well-preserved fauna are located on the northern Franconian Alb near Wattendorf, respectively on the southwestern part of the Swabian Alb near Nusplingen. In addition to these sites in southern Germany, Upper Jurassic Plattenkalk deposits with a rich fossil assemblage are also known from southeastern France [21].

Although these sites are overall similar, some significant differences are noteworthy with respect to lithology, age and faunal composition. As outlined above, the deposits are all composed of fine-grained limestones, the so-called Plattenkalk, yet the amount of silica and mud as well as the thickness of the limestone layers is highly variable, likely reflecting slightly distinct depositional environments [20]. Moreover, the different fossil sites of the Solnhofen Archipelago belong to at least four distinct formations–from oldest to youngest, the Torleite (Malm Epsilon), Geisental (Malm Zeta 1), Painten (Malm Zeta 1), Altmühltal (Malm Zeta 2) and Mörnsheim (Malm Zeta 3) formations–ranging in age from the upper Kimmeridgian to the lower Tithonian [22–24]. During the time of deposition in the latest Jurassic, southern Germany were situated in a shallow and tropical sea, with small and larger islands located nearby. In this setting, the ‘Plattenkalk’ was likely deposited in locally restricted basins or ‘Wannen’ that were bordered by sponge bioherms and thus were separated from the open sea; the resulting stagnant water conditions coupled with the tropical climate caused the depletion of oxygen and hypersaline conditions near the ground, which probably lead, together with rapid sedimentation during periodic storm events, to the exceptional preservation of the fossils [21].

The new *Solnhofia* specimen was found in the quarry of the Rygol Company close to Painten. The quarry is situated in the northern part of the ‘Paintener Wanne’, a locally restricted basin covering an area of approximately 15 x 12 km [7]. The specimen was found at the bottom of the exposed section in the ‘Kieselplattenkalk’, a 5.9 m thick package of laminated, fine-grained, silicified limestone intercalated with graded turbidite horizons consisting of carbonate debris [7, 25]. Stratigraphically, the ‘Kieselplattenkalk’ of Painten belongs to the Arnstorf Member of the Torleite Formation, which has been assigned to the *Hybnoticeras beckeri* ammonite Zone and *Lithacoceras ulmense* Subzone, corresponding to a latest Kimmeridgian age [22, 24]. Although the quarry has been operated since the 1950s, fossils have only come to light after 2001 when systematic excavations started, and over the last 20 years, a rich and diverse fossil assemblage has been unearthed comprising abundant plant remains, invertebrates (including sponges, corals, crinoids, brachiopods, ammonites, coleoids, gastropods, crustaceans and echinoderms) and vertebrates [7]. Among the latter, fishes are by far the most common group, being represented by actinopterygians, chondrichthyans, and coelacanths [7]. Fossil reptiles are comparatively abundant and include rhynchocephalians, ichthyosaurs, turtles, the thalattosuchians *Cricosaurus albersdoerferi* and *Dakosaurus* sp., atoposaurid neosuchians, *Pterodactylus antiquus* and other pterosaurs, and the theropod dinosaur *Sciurumimus albersdoerferi* [7–11, 26, 27]. Most of these specimens, however, have not been described in detail yet and several probably represent new taxa. The abundance of plants and terrestrial vertebrates indicates that islands must have been located nearby, while the presence of coleoids demonstrate that the local basin (‘Paintener Wanne’) was connected to the open sea [28].

## Material and methods

The specimen is permanently housed and accessible in the collection of the Dinosaurier Museum Altmühltal in Denkendorf, Bavaria, Germany (DMA) under the collection number DMA-JP-2004/005. In addition, the owner (RA) of the specimen guarantees by contract that it will always be available for science and has to be kept in a publicly accessible collection. A copy of this contract is available upon request. Phylogenetic nomenclature in this paper follows Joyce et al. (2021) [29].

### Systematic palaeontology

*Testudinata* Klein, 1760 [30] (sensu Joyce et al., 2020 [31])

*Pan-Cryptodira* Joyce et al., 2004 [32] (sensu Joyce et al., 2020 [33])

*Thalassochelydia* Anquetin et al., 2017 [2] (sensu Joyce et al., 2021 [29])

*Sandownidae* Tong and Meylan, 2013 [34] (sensu Joyce et al., 2021 [29])

### Remarks

As outlined above, *Solnhofia parsonsi* has usually been referred to the “Eurysternidae” in the past [1, 2, 13]. The monophyly of Eurysternidae is yet to be rigorously tested and *Solnhofia parsonsi* is more recently referred to the clade *Thalassochelydia* [2, 17, 18, 35] with some works even placing it into the less inclusive clade *Sandownidae* as part of *Thalassochelydia* [6, 19, 29], a taxonomy here followed. *Thalassochelydia* have typically been regarded as basal pan-cryptodirans, although they were recently also recovered as stem-testudines, stem-pleurodires, or stem-chelonioideans [2, 6, 17, 18, 35–38].

*Solnhofia* Gaffney, 1975 [12]

### Type species

*Solnhofia parsonsi* Gaffney, 1975 [12]

### Included valid species

*Solnhofia parsonsi* Gaffney, 1975 [12] and *Solnhofia brachyrhyncha* Anquetin and Püntener, 2020 [4].

*Solnhofia parsonsi* Gaffney, 1975 [12]

### Holotype

TM 4023, an almost complete and well-preserved skull and mandible.

### Referred material

DMA-JP-2004/005, a complete skull and skeleton preserved in articulation.

### Locality

Rygol Quarry near Painten, Niederbayern, Bavaria, Germany.

### Stratum

Torleite Formation, Beckeri zone, Ulmense subzone, upper Kimmeridgian, Upper Jurassic.

## Description

The new specimen of *Solnhofia*, DMA-JP-2004/005, comprises a complete skull and skeleton preserved in articulation (Fig 1). In addition to being complete, the specimen is very well preserved with most sutures and scute impressions well visible. However, the specimen is only exposed in dorsal view and consequently, the mandible and ventral aspect of the skull as well as the plastron cannot be examined. Of the axial skeleton, only three poorly preserved cervical vertebrae and several disarticulated caudal vertebrae are visible.

### Skull

**Fig 2:**
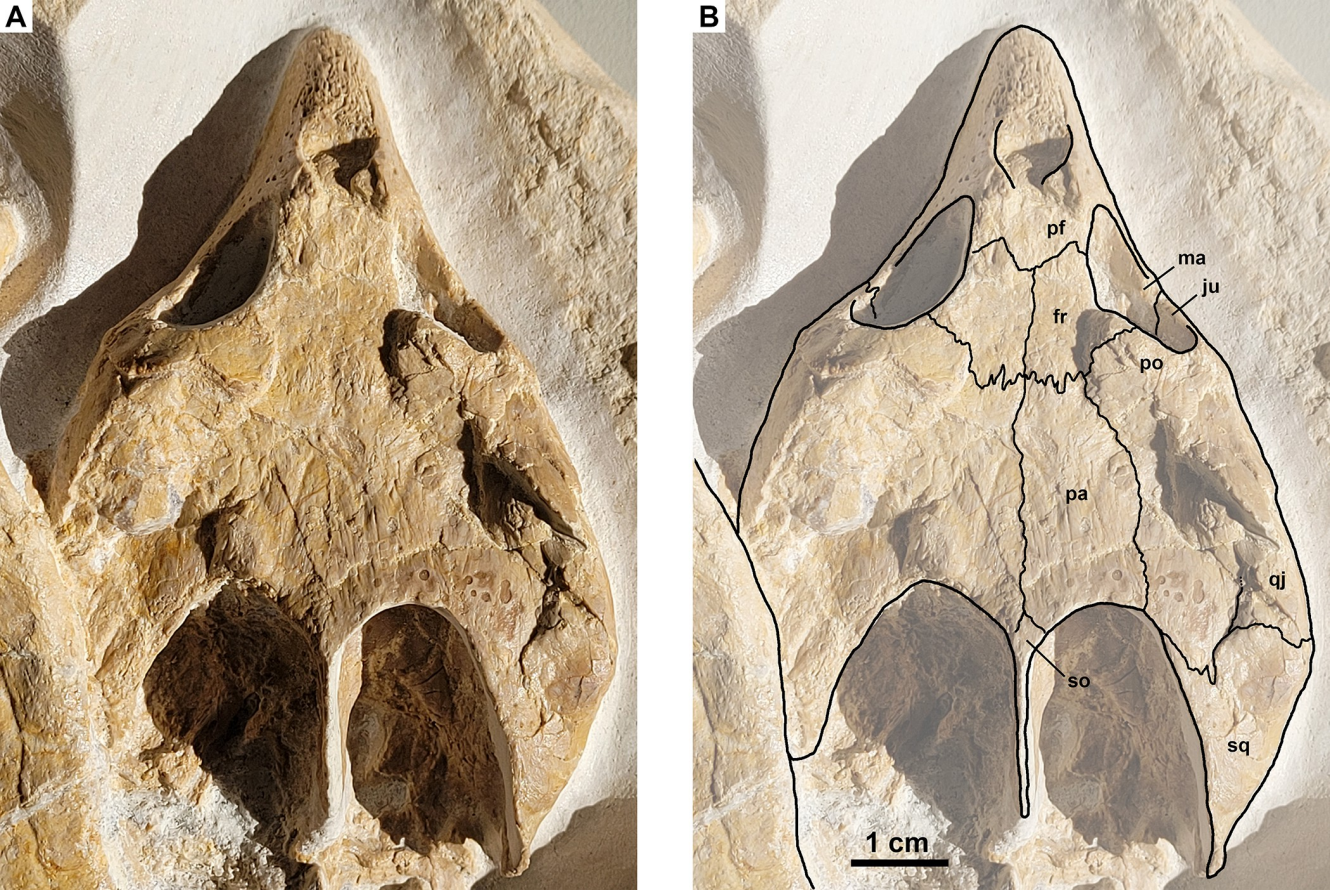
*Solnhofia parsonsi*, DMA-JP-2004/005, from the Upper Jurassic (Kimmeridgian) Torleite Formation of Painten. Detail photograph (A) and drawing (B) of the skull. Both to the same scale. Abbreviations: fr, frontal; ju, jugal; ma, maxilla; pa, parietal; pf, prefrontal; po, postorbital; qj, quadratojugal; so, supraoccipital; sq, squamosal.

The skull is complete and well preserved (Fig 2), only a small area on the posterolateral aspect is slightly damaged due to diagenetic compression. It is well exposed in dorsal view, although the left posterior part is partially covered by the carapace. In lateral view, the skull is exposed until the level of the oral margin anteriorly and the cavum tympani posteriorly; the more ventral parts are still encased in the rock matrix and thus are not visible. The skull has a triangular outline in dorsal view with a relatively elongated snout region in front of the orbits and a mediolaterally wide temporal region. Moreover, it exhibits large temporal emarginations that expose the posterodorsal aspect of the otic region. The skull has length of 9.2 cm and a width of more than 8 cm near the posterior margin, although the exact width cannot be measure due to the left posterior part being slightly covered by the carapace. Compared to the size of the carapace, the skull is very large, reaching approximately 40% of the carapace length. Overall, the skull bones are heavily fused to each other, making the recognition of many sutures in this specimen difficult to impossible. In addition, the skull shows almost no scute impressions.

**Fig 3:**
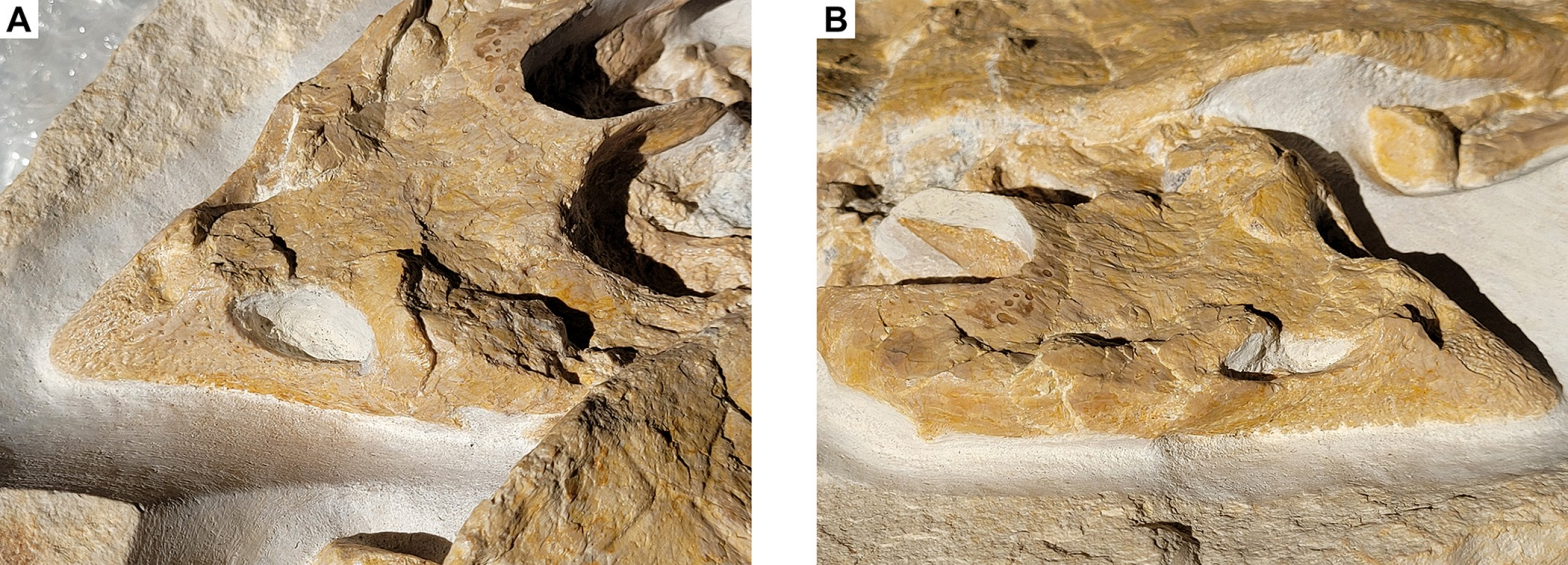
*Solnhofia parsonsi*, DMA-JP-2004/005, from the Upper Jurassic (Kimmeridgian) Torleite Formation of Painten. Detail photograph of the skull in left anterolateral (A) and right lateral view (B).

The anterior part of the skull forms an elongated and pointed snout region. The anterior-most part of the snout has a rugosely textured surface that extends posteriorly up to the external nares, indicating the presence of an extensive keratinous beak. The external nares are oval and slightly wider than high, although the opening itself is filled with bone and rock matrix. Posteriorly, the snout region rises markedly (Fig 3). In the snout region, no sutures between the individual bones are discernible. The skull roof is relatively flat and widens posteriorly. On the skull roof, the following sutures are visible: between the frontals and the prefrontals, the frontals and the parietals, the frontals and the postorbitals, the right parietal and the postorbital, the parietals and the supraoccipital, the squamosal and the postorbital respectively the quadratojugal as well as the median suture between the left and right frontals respectively parietals. No other sutures are discernible on the dorsal and lateral aspects of the skull. Nasals, reported for SM 137 [12], JM SCHA 70 [13] and MJSN BAN001-2.1 [4], are not discernible.

The frontals are roughly trapezoidal in outline with a triangular projection anteriorly. The suture between the left and right frontal is straight and extends anteroposteriorly. The frontals form a large portion of the orbital roof and posteriorly extend well beyond the orbits. The suture between the frontals and the postorbitals extends posteromedially, whereas the suture between the frontals and the parietals extends mediolaterally. The parietals are anteroposteriorly elongated and reach the temporal emargination posteriorly. Posteromedially, the parietals contact the supraoccipital, which forms a spur-like posterior projection that separates the left and right temporal emargination. The temporal emarginations in turn, are ellipsoidal in dorsal view and relatively large, having a length of approximately one third that of the skull length. The squamosal forms the posterolateral part of the skull and has a triangular shape, contacting the quadratojugal along a mediolaterally extending suture. Moreover, within the posteroventral part of the orbit, the suture between the maxilla and the jugal is visible extending posteromedially. The orbits are relatively large, oval to round in morphology and face anterolaterally.

### Carapacial bones

**Fig 4:**
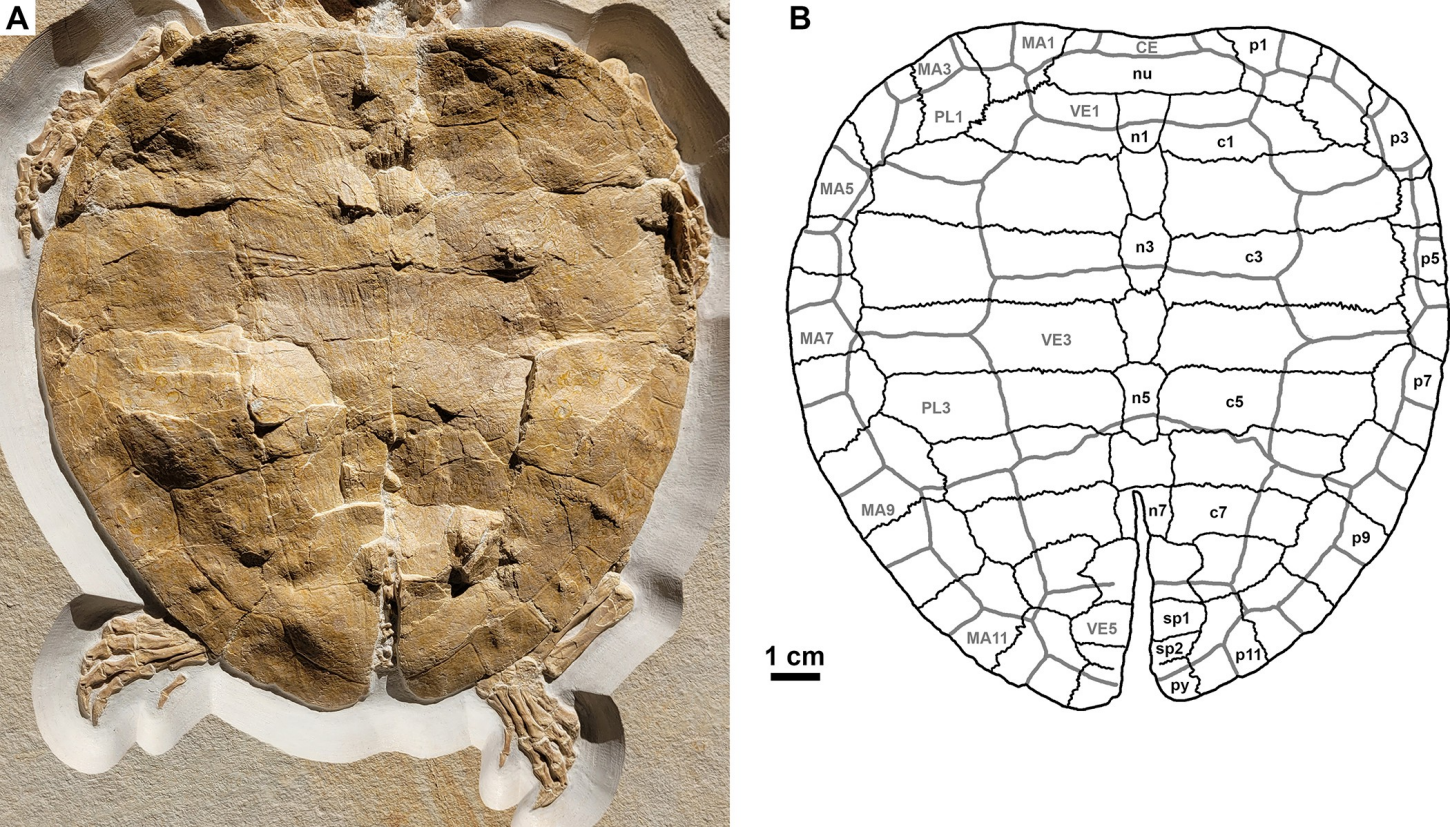
*Solnhofia parsonsi*, DMA-JP-2004/005, from the Upper Jurassic (Kimmeridgian) Torleite Formation of Painten. Detail photograph (A) and drawing (B) of the carapace. Both to the same scale. Abbreviations: c, costal; CE, cervical scute; MA, marginal scute; n, neural; nu, nuchal; p, peripheral; PL, pleural scute; py, pygal; sp, suprapygal; VE, vertebral scute.

The carapace has an anteroposterior length of 23 cm and a maximum mediolateral width of 22 cm at the level of peripheral 6 (Fig 4). Overall, the carapace has an oval to slightly heart-shaped outline. The carapace consists of the nuchal, eight neurals, two suprapygals, the pygal, eight costals and eleven peripherals. Due to taphonomic compression, the posterior part of the carapace is slightly split along the midline across neurals seven and eight. Aside from this break, a small portion in the anterolateral region of the carapace is slightly distorted affecting mainly peripherals 4 and 5. The surface of the shell is generally smooth, although several shallow subparallel grooves are present on the anterior half of the carapace, radiating from the midline.

The nuchal is a trapezoidal element and wider mediolaterally than long anteroposteriorly with a width to length ratio of approximately 3:1. The anterior margin of the nuchal is slightly concave, resulting in a weakly developed and relatively wide nuchal emargination. The nuchal is sutured to peripheral 1 laterally along a straight to slightly convex suture that extends posterolaterally. The neural series consists of eight elements, most of which have a rather elongated morphology. Neural 1 is quadrangular, slightly longer than wide and has a convex posterior margin. Neurals 2–5 are more elongated and have a hexagonal, coffin-shaped outline. Neurals 6 and 7 differ from the more anteriorly placed neurals in having an almost square-like morphology, being as wide as long or even wider than long. Neural 8, again, has a different morphology, being star-shaped and wider than long. Two suprapygals and one pygal element are present. The two suprapygals have a roughly semi-circular morphology, being much wider than long, and meet one another along a straight, mediolaterally extending suture. The pygal is trapezoidal element that is much wider than long and widens posteriorly. The anterior margin, where the pygal is sutured to suprapygal 2, is slightly concave, whereas the posterior margin is slightly convex.

Eight costals are present on the right and on the left side of the carapace. Costal 1 has a roughly trapezoidal outline and is about twice as wide as long. It is sutured to the nuchal anteriorly, to neural 1 medially, to neural 2 posteromedially, to peripheral 1 and 2 laterally, and to costal 2 posteriorly. In contrast tocostal 1, costals 2–7 are rectangular in outline and much wider than long. Additionally, they are usually sutured to the adjacent costals anteriorly and posteriorly, to two consecutive neurals medially, and to two peripherals laterally. The only exceptions to this pattern among costals 2–7 are costal 2, which is sutured to three peripherals laterally, and costal 6, which is sutured to only one neural medially. Costal 8 is much shorter mediolaterally than the more anteriorly placed costals and accordingly has a triangular to trapezoidal outline, being only slightly wider than long. It is sutured to costal 7 anteriorly, to neural 8 medially, to suprapygal 1 posteromedially, to peripheral 10 laterally and to peripheral 11 posterolaterally.

Along the lateral margin of the carapace, eleven peripherals are present. The peripherals are blocky elements with a trapezoidal to rectangular outline and are nearly as wide as long. They decrease in size from peripheral 1 until peripheral 5, then increase in size again until peripheral 8, before they decrease in size again. Peripheral 1 is sutured to the nuchal medially, to the first costal posteriorly, and to the second peripheral laterally. Peripherals 2–10 are sutured to the adjacent peripherals anteriorly and posteriorly as well as to the costals medially. Aside from peripherals 1, 3 and 11, which are sutured to only one costal, all peripherals make contact with two costals. Peripheral 11 contacts the eighth costal anteriorly, the two suprapygals and the pygal medially and the tenth peripheral anterolaterally. Costo-peripheral fontanelles are absent.

### Carapacial scutes

The carapace exhibits impressions of the cervical scute, five vertebral scutes, four pairs of pleural scutes and twelve pairs of marginal scutes (Fig 4). The cervical scute was trapezoidal in outline and relatively small, covering only the anterior part of the nuchal. The cervical scute contacted the marginals 1 laterally and vertebral 1 posteriorly. Vertebral scute 1 was much larger than the cervical, had an oval to hexagonal outline and was much wider than long. It was bordered by the cervical anteriorly, marginal 1 anterolaterally, pleural 1 laterally and vertebral 2 posteriorly. Vertebrals 2 and 3 were similar in size, had a roughly hexagonal shape and they too, were wider than long, although both were proportionately longer anteroposteriorly than vertebral 1. Vertebral 4 differed from the more anteriorly placed vertebrals in that it had a more trapezoidal to rectangular shape and in being proportionately much longer anteroposteriorly. Vertebral 4 was bordered almost exclusively by pleural scute 4, contacting pleural 3 only at its anterolateral corner. Vertebral 5 was much smaller than vertebrals 2–4 and trapezoidal to oval in shape. It was bordered by vertebral 4 anteriorly, marginal 12 posteriorly, and laterally, by the pleural 4 and marginal 11. Marginals 2–11 were restricted to the peripherals, while marginal 1 overlapped also the nuchal and marginal 12 overlapped also the pygal.

### Appendicular skeleton

**Fig 5:**
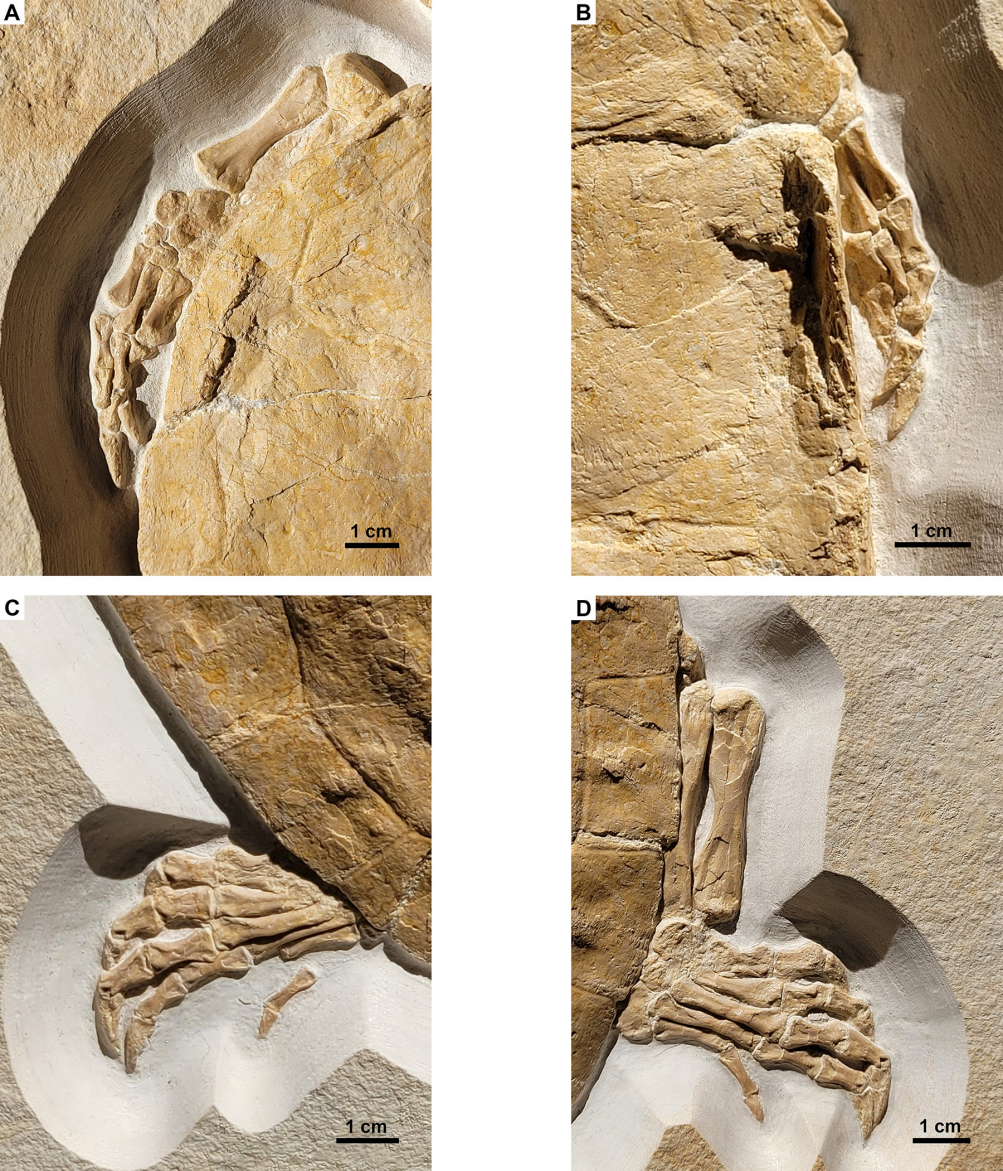
*Solnhofia parsonsi*, DMA-JP-2004/005, from the Upper Jurassic (Kimmeridgian) Torleite Formation of Painten. Detail photograph of the left forelimb (A), the right forelimb (B), the left hindlimb (C) and right hind limb (D). Photos are not to the same scale.

All four limbs are preserved in the *Solnhofia* specimen from Painten, although none of them is completely exposed, as the proximal elements are hidden in the shell (Fig 5). In addition, the limbs are, just like the rest of the skeleton, only exposed in dorsal view because the ventral part of the specimen is still encased in sediment. The forelimbs are both exposed from the distal tips of the claws until the distal articular surface of the humeri proximally. Both radii is partially to completely hidden under the carapace margin. The ulna and radius are slightly distorted in the left forelimb, in which the ulna is lying lateral to the radius. The radius is straight, slightly bowed and longer than the ulna. The ulna is more massively built than the radius and has markedly expanded proximal and distal articular surfaces. The proximal carpals comprise two block-like elements that lie directly distal to the ulna, the intermedium medially and the slightly larger ulnare laterally. Of the distal carpals, only three disc-shaped elements are visible on the slightly better exposed right forelimb. Three metacarpals are discernible for each hand, mc I–III, of which mc I is the shortest, while the remaining two are subequal in length. The phalanges are relatively short and each digit terminates with a well-developed claw. The phalangeal formula for the manus is 2-3-3-?-?.

The hind limbs are both visible, although the right one is much better exposed than the left one, in which only the pes (i.e. metatarsals and phalanges) is discernible. In the right hind limb, the tibia and fibula, some of the tarsals and the complete pes are visible. The tibia is slightly more robust but shorter than the fibula. Both elements are relatively straight and have a rod-like morphology with expanded articular surfaces proximally and distally. The tarsals are partially hidden under the carapace margin and are imperfectly preserved. Only the large block-like proximal tarsal and some poorly preserved disc-shaped distal tarsals are discernible. Distally, the metatarsals are well preserved. The first four metatarsals are rod-like elements with slightly expanded articular surfaces proximally and distally. The first metatarsal is the shortest, followed by the second metatarsal. Metatarsals III and IV are the longest and are subequal in length. In contrast to this, metatarsal V is a plate-like element that is distally hidden under the carapace and thus not fully visible in the right hind limb. The phalanges are relatively short and each digit terminates with a well-developed claw. The phalangeal formula for the pes is 2-3-3-3-3.

## Comparison

The specimen described herein, DMA-JP-2004/005, is clearly referable to the genus *Solnhofia* based on the presence of the following combination of characters: an enlarged skull, limited temporal emargination, reduced to absent costo-peripheral fontanelles (for a complete diagnosis of the genus, see above). In the following comparison, DMA-JP-2004/005 is compared to all published specimens of *S*. *parsonsi*. These include the holotype skull TM 4023 and the referred isolated skull NMS 8741 (previously catalogued as NMS 137, [4, 12]), both of which were described by Gaffney (1975) [12], as well as the skeleton JM SCHA 70, described by Joyce (2000) [13]. In addition, we compare DMA-JP-2004/005 to *S*. *brachyrhyncha*, which is known from a two skulls and numerous isolated postcranial elements [4].

### Skull

The skull of DMA-JP-2004/005 closely resembles that of TM 4023, NMS 8741 and JM SCHA 70 in having an elongated, roughly triangular outline in dorsal view with a long and pointy snout region. In addition, DMA-JP-2004/005 and JM SCHA 70 have a very similar skull to carapace length ratio of approximately 40%. In all four specimens, the anterior-most part of the snout has a rugosely textured surface. The external nares are oval and the orbits are relatively large and face anterolaterally. The skull roof is relatively flat and widens posteriorly. The temporal emarginations of DMA-JP-2004/005 seem to be proportionately somewhat larger than those of TM 4023 and NMS 8741, although the posterior region of the skull surrounding the temporal emarginations is only incompletely preserved and damaged in the latter two specimens. In JM SCHA 70, the posterior and lateral portions of the skull are badly damaged dorsally.

Concerning the sutures of the skull bones, no apparent differences are present between the four specimens of *S*. *parsonsi*, although it should be noted that many sutures are not clearly discernible in all of them. The holotype skull of *S*. *brachyrhyncha* (MJSN BAN001-2.1) is only incompletely preserved as well as heavily deformed and crushed, thus making a comparison difficult. It differs from all specimens of *S*. *parsonsi* in having a much shorter snout region; however, the anterior portion of MJSN BAN001-2.1 is not complete and thus the size and shape of the snout is difficult to assess. Aside from the shorter snout, MJSN BAN001-2.1 has a proportionately much larger frontal than DMA-JP-2004/005, TM 4023, NMS 8741 and JM SCHA 70, which anteriorly extends almost until the margin of the orbit.

### Carapacial bones

The overall shape of the carapace in DMA-JP-2004/005 resembles that of JM SCHA 70 in being oval and slightly longer than wide. However, the shape of the carapace differs in that the widest part is placed more anteriorly in DMA-JP-2004/005, approximately at the level of peripheral 6 and costal 3, whereas the widest part in JM SCHA 70 is placed at the level of peripheral 8 and costal 6. Because of this difference in the shape, the posterior part of the carapace is also narrower mediolaterally in DMA-JP-2004/005 with a slightly pointed tip. The narrower nuchal and the larger peripherals 1 to 2 of DMA-JP-2004/005 makes the anterior part of the carapace wider and longer compared to JM SCHA 70. Another important difference between DMA-JP-2004/005 on the one hand, and JM SCHA 70 and *S*. *brachyrhyncha* on the other is the lack of carapacial fontanelles in the former.

Neurals 6–8 are markedly different between DMA-JP-2004/005 and JM SCHA 70. In the latter, neural 6 is elongated, hexagonal and coffin-shaped (i.e., shaped like the more anterior neurals) but neurals 7–8 are proportionately much shorter, being roughly as wide as long. In DMA-JP-2004/005, neurals 6–7 are rectangular and roughly as wide as long, and neural 8 is star-shaped and much larger than neural 6–7, being the widest in the series. The suprapygals are only imperfectly preserved in JM SCHA 70 and thus cannot be compared to those of DMA-JP-2004/005, although suprapygal 1 seems to have been proportionately larger. In addition, three suprapygals seem to be present in JM SCHA 70, as opposed to two suprapygals in DMA-JP-2004/005, although this is difficult to ascertain due to the imperfect preservation of JM SCHA 70. In *S*. *brachyrhyncha*, neither the nuchal nor the pygal or suprapygals are known. In addition, only isolated neurals have been referred to this species so far.

Costal 1 is mediolaterally shorter in DMA-JP-2004/005 than in both JM SCHA 70 and *S*. *brachyrhyncha*. Moreover, costal 1 only contacts peripheral 1 and 2 in DMA-JP-2004/005, whereas it contacts peripherals 1–3 in JM SCHA 70 and *S*. *brachyrhyncha*. Furthermore, costal 1 and 2 only contact neural 1 and neural 2, respectively in JM SCHA 70, whereas in DMA-JP-2004/005, costal 1 contacts neurals 1–2 and costal 2 contacts neural 2–3. DMA-JP-2004/005 further differs from JM SCHA 70 in that costal 6 only contacts neural 6, whereas in JM SCHA 70, costal 6 contacts both neural 6 and 7. Based on these isolated elements, however, it is apparent that costals 2–5 had no sutural lateral contact with the peripherals in *S*. *brachyrhyncha* and thus it differs from DMA-JP-2004/005 (in which all costals are sutured to the peripherals) and JM SCHA 70 (in which only costals 2–4 have no lateral sutural contact to the peripherals).

### Carapacial scutes

Regarding the carapacial scutes, several important differences are present between the different *Solnhofia* specimens, mostly concerning their arrangement, size and morphology. The cervical scute was relatively large in DMA-JP-2004/005 and covered approximately two-thirds of the anterior width of the nuchal, whereas the cervical scute in JM SCHA 70 was much smaller covering only one-third of the nuchal anteriorly. Moreover, the cervical scute was also somewhat more elongated anteroposteriorly in DMA-JP-2004/005 than in JM SCHA 70. Vertebral I, in contrast, was narrower in DMA-JP-2004/005 than in JM SCHA 70. In the latter, vertebral I covered the majority of peripheral 1 laterally and reached almost the posterior margin of costal 1 posteriorly, while in DMA-JP-2004/005 vertebral I only covered a small part of peripheral 1 laterally and a much smaller portion of costal 1 posteriorly. The size of vertebral I of *S*. *brachyrhyncha* seems to have been intermediate in this regard.

Vertebrals 1–3 of DMA-JP-2004/005 differed greatly from those of JM SCHA 70 and *S*. *brachyrhyncha* in that they were much narrower mediolaterally covering only approximately half of the costal width, as opposed to covering nearly 80% in JM SCHA 70. In addition, the sulcus between vertebral 3 and 4 is curved and runs across costal 5 and 6 in DMA-JP-2004/005, whereas it is straight and runs only across costal 5 only in both JM SCHA 70 and *S*. *brachyrhyncha*. The positions of the other vertebrals (i.e., vertebral I, II and V) relative to the carapacial bones were largely similar in DMA-JP-2004/005, JM SCHA 70, and *S*. *brachyrhyncha* with vertebral I covering the nuchal, peripheral 1 and costal 1, vertebral II covering costal 1–3, and vertebral V covering costal 8, the two suprapygals, the pygal and peripherals 10 and 11. Additionally, DMA-JP-2004/005 resembles JM SCHA 70 in that the sulci between vertebrals I and II, vertebrals II and III, as well as vertebrals IV and V are straight and only extend mediolaterally.

As a result of the much narrower vertebrals, the pleurals were proportionately much wider in DMA-JP-2004/005 than in JM SCHA 70 and *S*. *brachyrhyncha*. DMA-JP-2004/005 further differs from JM SCHA 70 and *S*. *brachyrhyncha*, in the position of pleural 1 relative to the carapacial bones. In DMA-JP-2004/005, pleural I covered only peripherals 1–3 laterally, while in JM SCHA 70 and *S*. *brachyrhyncha*, pleural I covered peripherals 1–4 laterally. Medially, pleural I covered costal 1 and 2 in DMA-JP-2004/005, JM SCHA 70 and *S*. *brachyrhyncha*. Pleural IV covered four peripherals in DMA-JP-2004/005, but only three in JM SCHA 70. The marginal scutes were rather similar in DMA-JP-2004/005, JM SCHA 70, and *S*. *brachyrhyncha*, although they were somewhat wider mediolaterally in DMA-JP-2004/005 than in both JM SCHA 70 and *S*. *brachyrhyncha*.

## Discussion and conclusion

### Taxonomy of DMA-JP-2004/005

A new specimen of the pan-cryptodiran turtle *Solnhofia parsonsi* from the Plattenkalk deposits of Painten (Torleite Formation, Kimmeridgian) is described. It is only the second described specimen preserving both the skull and the postcranium in articulation. Whereas the first of these, JM SCHA 70, is extremely well preserved in ventral view, DMA-JP-2004/005 exhibits a much better-preserved carapace in dorsal view. The specimen DMA-JP-2004/005 can be confidently referred to *Solnhofia parsonsi* based on the presence of the following combination of characters: an enlarged skull, an elongated snout, moderate temporal emargination, reduced to absent costo-peripheral fontanelles, a pentagonal carapace, a broad nuchal that forms a wide nuchal notch, broad posterior peripherals, and a large pygal (for the complete diagnosis, see [4]). The carapace of DMA-JP-2004/005 differs in several respects from JM SCHA 70, the only other currently published specimen of *S*. *parsonsi* preserving the postcranium. Most of these can be attributed to ontogenetic change in carapace and scute proportions (e.g., [39]) as DMA-JP-2004/005 is a larger and therefore presumably older individual than JM SCHA: (i) the carapace shape (i.e. carapace being wider more anteriorly); (ii) the absence of carapacial fontanelles, (iii) costal 1 contacts only peripheral 1 and 2, (iv) narrower vertebral scutes (probably as a result, the sulcus between vertebral III and IV is curved anteriorly and runs across costal 5 and 6), (v) pleural I covering only peripherals I–III and not overlapping onto peripheral IV. A star-shaped and very large neural 8 in DMA-JP-2004/005 may represent intraspecific variability, possibly an abnormality.

### Temporal distribution of *Solnhofia parsonsi*

Specimen DMA-JP-2004/005 from Painten represents the first occurrence of *S*. *parsonsi* from the Upper Jurassic (Kimmeridgian) Torleite Formation (see above). The holotype skull TM 4023 described by Gaffney (1975) [12] probably originates from the Solnhofen area and, based on this, has been assumed to be from the Solnhofen Formation [2], which has been estimated to be early Tithonian in age [22, 23], although the exact horizon and place of origin is unknown. The referred skull NMS 8741, also described by Gaffney (1975) [12], probably comes from the Kimmeridgian (*Pseudomutabilis* ammonite zone) of Solothurn, Switzerland [12]; however, as for the holotype skull, the exact place of origin is unknown. The referred skeleton JM SCHA 70 described by Joyce (2000) [13] was recovered from the Plattenkalk deposits of Schamhaupten that belong to the Painten Formation [40] and were estimated to be uppermost Kimmeridgian in age [22, 23]. Purported finds of *S*. *parsonsi* from the laminated limestones of Eastern France come from the Kimmeridgian of Labastide-Murat and the Tithonian of Canjuers [15, 16], but the taxonomic affinities of these specimens and the assignment to *S*. *parsonsi* have been questioned [2]. Therefore, DMA-JP-2004/005 represents only the second undisputed specimen of *S*. *parsonsi* (besides JM SCHA 70 from Schamhaupten), for which detailed information on its locality and stratigraphic position are known.

### Relationship between limb taphonomy and habitat ecology in fossil turtles

DMA-JP-2004/005 is the first described specimen of *Solnhofia* preserving largely complete and articulated fore- and hind limbs and thus offers new insights into the locomotory behaviour of this taxon. The limbs of DMA-JP-2004/005 (especially the forelimbs) are preserved adducted with the digits being stacked on top of one another (Fig 5). Based on the study of Joyce et al. (2021) [6], this implies that *S*. *parsonsi* lacked stiffened paddles as otherwise present in more pelagic marine turtles. Joyce et al. (2021) [6] demonstrated that in laminated deposits, freshwater turtle flippers typically preserve with digits adducted whereas those of unambiguous marine turtles in a splayed-out position. The authors pointed out that the splayed-out position is due to the presence of rigid paddles with flattened digits and can be used to infer marine ecology of fossil taxa. Less explicitly, they furthermore implied that the adducted position predicts freshwater habitat: “Turtles from the Late Jurassic platy limestones of Europe show both poses, which supports the notion that both marine and freshwater aquatic turtles are deposited in the basins” [6]. We agree that a splayed-out position is reasonably expected to be more common in taxa with stiffened paddles, i.e., taxa that all happen to be marine. On the other hand, marine lifestyle is not necessarily correlated with stiffened paddles as the latter is an advanced swimming adaptation (e.g., [41]), plausibly absent in less capable swimmers preferring nearshore marine habitats. Fossil autopodia of nearshore marine taxa are expected to preserve in an adducted pose as long as they are not stiffened by scales and/or the loss of interphalangeal joints. Evidence for a flexible flipper alone should therefore not be interpreted as evidence for freshwater lifestyle.

While there are no living sea turtles with flexible flippers, extinct nearshore marine bothremydid pleurodires exemplify our point: many of them are interpreted as costal turtles on the basis of widespread occurrence in shallow marine deposits and the presence of a deep skull [42] whilst they had flexible flippers judging from their interphalangeal articulations [43]. Although the examples of fossil turtles with an adducted pose listed in Joyce et al. (2021) [6] to demonstrate a pattern indeed consistently come from freshwater deposits, there is a notable exception: a taxon from the Jurassic marine Plattenkalk of Germany with otherwise unknown habitat preference (*Parachelys* sp.). In fact, on the basis of movable interphalangeal joints or adducted preservation of hands alone, a turtle fossil from nearshore marine deposits (such as the Jurassic Plattenkalk of Germany) can be interpreted as freshwater (allochthonous) or costal (autochthonous) in ecology with equal confidence. A combination of further anatomical data (e.g., the validation of spaces for salt glands, plastral architecture), as well as taphonomic or occurrence data is necessary to reconstruct habitat preference and to avoid circular reasoning. We realize that Joyce et al. (2021) [6] may not have intended to explicitly establish correlation between taphonomy and freshwater ecology but clarifying this point may nevertheless help avoiding confusion.

### Habitat ecology of *Solnhofia parsonsi*

Previously, it has been suggested that *S*. *parsonsi* had a fully marine lifestyle based on its widespread occurrence in numerous shallow marine basins [44]. This is further supported by the presence of sexual dimorphism in tail length otherwise particularly pronounced in sea turtles among extant taxa (with females possessing considerably shorter tails, [45, 46]). Through a crack in the posterior portion of the carapace, DMA-JP-2004/005 reveals tiny caudal vertebrae below the carapace indicative of a short tail. This condition is similar to that of JM SCHA 70 but in marked contrast to the longer tail reaching well beyond the carapace in the two specimens on display at BMM, strongly suggesting sexual dimorphism (DMA-JP-2004/005 and JM SCHA 70 being females). Sexual dimorphism in tail length has been previously reported in other thalassochelydian turtles [5, 6]. With its relatively short flippers, movable interphalangeal articulations on the digits, presence of a central plastral fontanelle, lack of costo-peripheral fontanelles, a posteriorly tapering carapace reminiscent of chelonioids and protostegids, and wide triturating surface *Solnhofia parsonsi* is best interpreted as a costal marine benthic feeder.
